# Supplementary material for: Non-Pharmacological Management of Gestational Diabetes Mellitus with a High Fasting Glycemic Parameter: A Hospital-Based Study in Vietnam
Source: J Clin Med. 2024 Oct 2;13(19):5895. doi: 10.3390/jcm13195895 (PMC11478153; doi:10.3390/jcm13195895)
Supplement: Supplementary file 1 [file jcm-13-05895-s001.zip › jcm-3082837-supplementary.pdf]

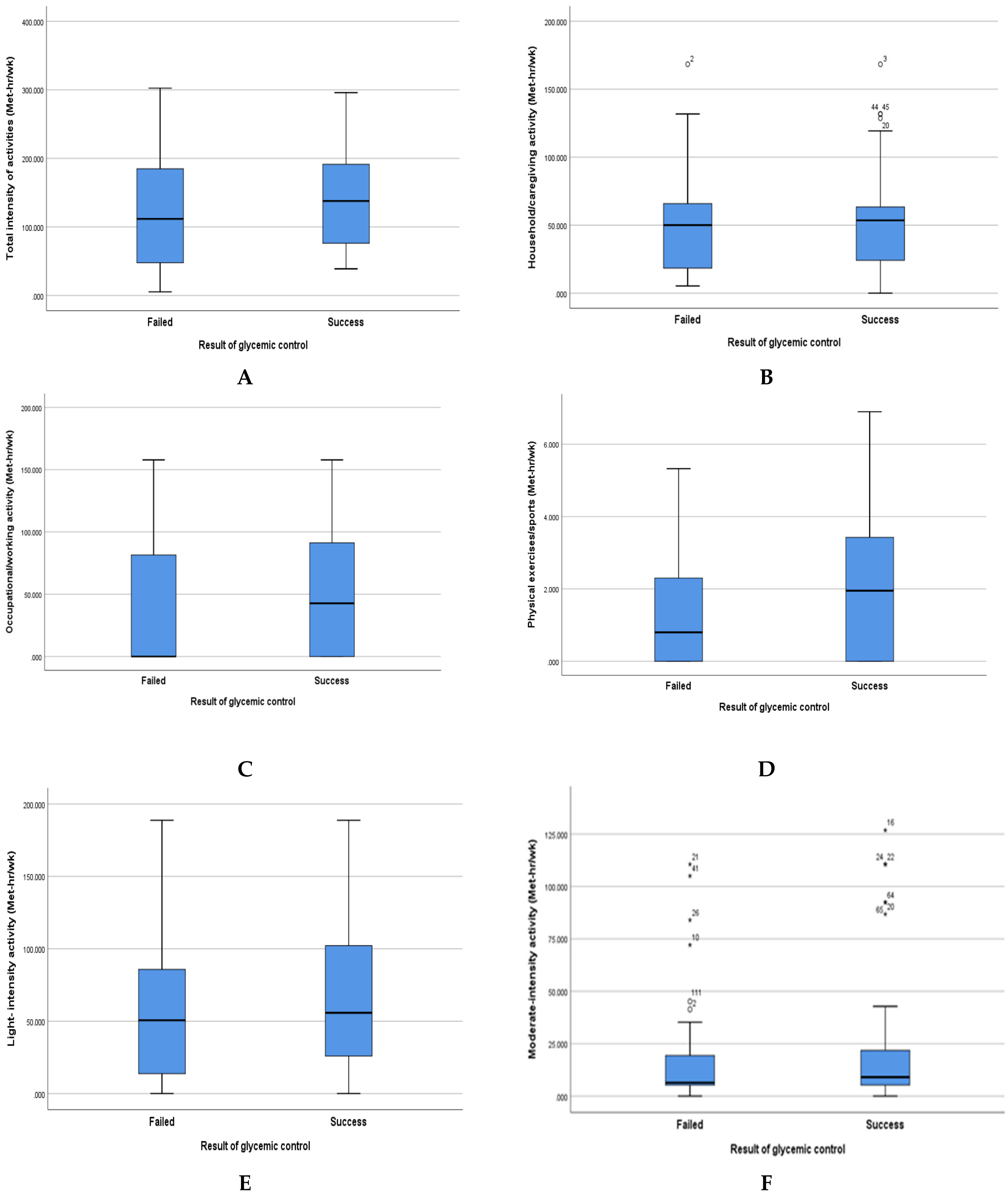

Supplementary Figure S1. Box plots show the difference of intensity (Met-hr-wk) between the failed and success groups of glycemic control regarding total activities (**A**), household/caregiving activities (**B**), occupation/working activities (**C**), physical exercise/ sport activities (**D**), light-intensity activities (**E**), and moderate-intensity activities (**F**).

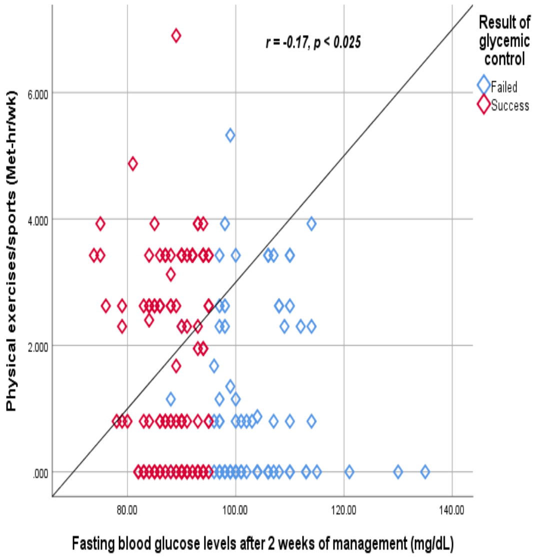

(A)

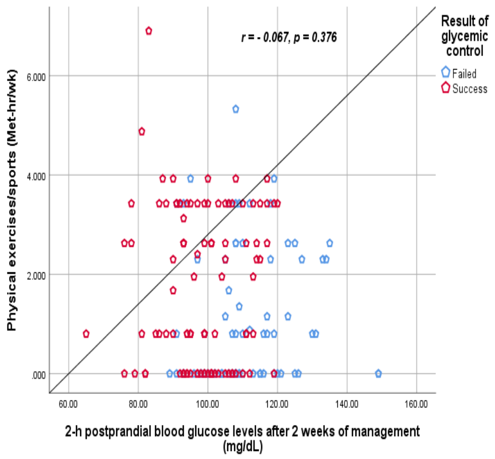

(B)

Supplementary Figure S2. Linear regression graphs show the correlation between physical exercise/sport activities and fasting blood glucose levels after 2 weeks of GDM management (**A**) and the correlation between physical exercise/sport activities and 2 h postprandial blood glucose levels after 2 weeks of GDM management (**B**).
